# The Latest View on the Mechanism of Ferroptosis and Its Research Progress in Spinal Cord Injury

**DOI:** 10.1155/2020/6375938

**Published:** 2020-08-28

**Authors:** Yixin Chen, Suixin Liu, Jianjun Li, Zhe Li, Jing Quan, Xinzhou Liu, Yinbo Tang, Bin Liu

**Affiliations:** ^1^Department of Rehabilitation, Xiangya Hospital of Central South University, Changsha, Hunan, China; ^2^Department of Spinal and Neural Function Reconstruction, China Rehabilitation Research Center, Beijing, China; ^3^Capital Medical University School of Rehabilitation Medicine, Beijing, China; ^4^Department of Spine Surgery, Hunan Provincial People's Hospital (The First Affiliated Hospital of Hunan Normal University), Changsha, Hunan, China

## Abstract

Ferroptosis is a recently identified nonapoptotic form of cell death whose major markers are iron dependence and accumulation of lipid reactive oxygen species, accompanied by morphological changes such as shrunken mitochondria and increased membrane density. It appears to contribute to the death of tumors, ischemia-reperfusion, acute renal failure, and nervous system diseases, among others. The generative mechanism of ferroptosis includes iron overloading, lipid peroxidation, and downstream execution, while the regulatory mechanism involves the glutathione/glutathione peroxidase 4 pathway, as well as the mevalonate pathway and the transsulfuration pathway. In-depth research has continuously developed and enriched knowledge on the mechanism by which ferroptosis occurs. In recent years, reports of the noninterchangeable role played by selenium in glutathione peroxidase 4 and its function in suppressing ferroptosis and the discovery of ferroptosis suppressor protein 1, identified as a ferroptosis resistance factor parallel to the glutathione peroxidase 4 pathway, have expanded and deepened our understanding of the mechanism by which ferroptosis works. Ferroptosis has been reported in spinal cord injury animal model experiments, and the inhibition of ferroptosis could promote the recovery of neurological function. Here, we review the latest studies on mechanism by which ferroptosis occurs, focusing on the ferroptosis execution and the contents related to selenium and ferroptosis suppressor protein 1. In addition, we summarize the current research status of ferroptosis in spinal cord injury. The aim of this review is to better understand the mechanisms by which ferroptosis occurs and its role in the pathophysiological process of spinal cord injury, so as to provide a new idea and frame of reference for further exploration.

## 1. Introduction

As an intrinsic phenomenon of metabolism, cell death serves as an exploratory topic in the cryobiology sector, such as embryonic development, homeostasis, neoplasia, and tissue renewing. Previous scholars have found there are different forms of cell death, including accidental cell death (ACD) and regulated cell death (RCD) [1, 2].

Among them, ACD is caused directly by physical, biological, or chemical factors that cause irresistible and irreversible damage to the plasma membrane or other components of the cell, such as organelles, rendering the cell unregulated and causing death. This type of death is often accompanied by the destruction of the cell membrane structure and obvious inflammation. ACD cannot be regulated by cells, so it is also called unregulated cell death [3]. RCD, also called programmed cell death (PCD), is a form of cell death under genetic and pharmacological regulation [4].

As early as 1956, scholars put forward the concept of PCD, which refers to a process of active death strictly regulated by cell signals, involving a series of gene activation, expression, and regulation. In addition to the earliest known apoptosis [5], other nonapoptotic forms of cell death have been discovered in recent years, including necroptosis [6], pyroptosis [7, 8], ferroptosis [9], entosis [10], netosis [11], parthanatos [12], lysosome-dependent cell death [13], autophagy-dependent cell death [14], alkaliptosis [15], and oxeiptosis [16].

Ferroptosis is a form of cell death marked by the requirement for iron and the accumulation of ROS. Early research on ferroptosis mainly focused on cancer [9, 17, 18], but further research identified ferroptosis in many other diseases, including ischemia-reperfusion injury [19], acute renal failure [20], Alzheimer's disease (AD) [21–23], Parkinson's disease (PD) [24, 25], and Huntington's disease (HD) [26–28]. In addition, the relationship between ferroptosis and acute central nervous system diseases, such as stroke [29, 30], traumatic brain injury [31–34], and SCI [35, 36], has been widely studied. The homeostasis of neurons depends on the balance between cell death induced by external or internal stress reactions and cell repair. Once the balance is broken, neural cells may face numerous diseases caused by cell death or over repair. This review focuses on the latest research on the mechanisms of ferroptosis in SCI, so as to provide a new idea and frame of reference for further exploration.

## 2. Ferroptosis and Its Mechanism

An essential nutrient for the human body, iron plays an active role in maintaining physiological functions. It not only participates in the composition of heme in hemoglobin but also serves as a coenzyme in various catalytic reactions. There are two states of iron in the human body, bound iron, and free iron. Bound iron exists mainly in the form of ferritin, such as hemoglobin and iron-sulfur nanoclusters, while free iron, also called nonbinding iron, mainly exists in heme or non-iron-sulfur nanoclusters. Free iron includes both ferrous iron and ferric iron. Ferrous iron has high reactivity and increases the cytotoxicity of ROS.

The proper level of iron maintains normal physiological function of the human body, but excessive free iron will cause harm to cells. As the donor of intracellular electrons, free iron undergoes redox transformation between ferrous iron and ferric iron. Ferrous iron generates (OH) hydroxyl radicals by means of the Fenton reaction, then induces oxidative stress and causes cell damage.

Ferroptosis was originally postulated in 2012 by Stockwell and Dixon, American scholars. They discovered the inhibiting effects of Erastin and RSL3 on human RAS mutation fibrosarcoma cell line HT-1080 and proved it was the result of ferroptosis. Different from the canonical cell death pathway (apoptosis, necrosis, autophagy, etc.), ferroptosis depends on intracellular iron and has morphological, biochemical, and genetic features [9]. Morphologically, ferroptosis shows shrinkage of mitochondria, increase of membrane density, and decrease or disappearance of mitochondrial cristae. Biochemically, it shows iron-dependent ROS and oxidative polyunsaturated fatty acids (PUFAs) increased. And genetically, it is mainly related to the genomic change of iron homeostasis and lipid peroxidation metabolism. Additionally, ferroptosis could be blocked by iron chelators such as deferoxamine (DFO) [9] and induced by Erastin and Sulfasalazine [37].

In essence, whether ferroptosis occurs is dependent on the balance between the ROS induced by iron enrichment and the antioxidant system that prevents lipid peroxidation. Once imbalance occurs, such as through excessive ROS production or decreased activity of the antioxidant system, it can trigger lipid peroxidation to damage the plasma membrane and induce iron death [38]. Certain concentrations of ROS can increase the repair of double-DNA strand breaks and promote cell survival by activating a series of reactions such as epidermal growth factor receptor (EGFR). Therefore, it is critical to the growth and development of the human body. However, excessive ROS will badly damage the biofilm, protein, and nucleic acid and lead to cell death.

**Figure 1:**
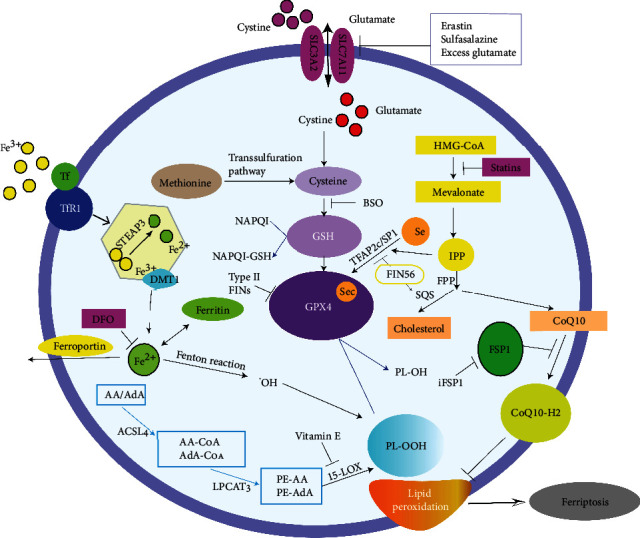
Summary of the latest mechanisms of ferroptosis, including occurrence mechanisms, regulatory mechanisms, and various inhibitors.

Ferroptosis is an iron-catalyzed lipid peroxidation process, the most notable feature of which is the formation of ROS, which can be induced by a nonenzymatic mechanism (Fenton reaction) or enzymatic mechanism (lipoxygenase) [39]. The main role of ROS is to cause oxidative damage to biofilms by targeting polyunsaturated fatty acids (PUFAs), while this process can be blocked by some lipophilic substances such as VE, Ferrostatin-1 (fer-1) and liproxstatin-1 (lip-1). Dixon and Stockwell [40] believed that the loss of lipid peroxide repair capacity by the phospholipidhydroperoxidase GPX4, the availability of redox active iron, and the oxidation of PUFA-containing phospholipids were the three most important markers of ferroptosis and that they were essential for ferroptosis to occur. A summary of the mechanisms by which ferroptosis occurs is shown in Figure 1.

### 2.1. The Mechanisms of Ferroptosis Occurrence

#### 2.1.1. Iron Metabolism Pathway

The first step in ferroptosis is the accumulation of iron. A large amount of free Fe^3+^ in the blood forms a complex with extracellular transferrin (Tf), which binds on transferrin receptor 1 (TFR1) on the cell membrane and transplants into the cell in the process of endocytosis [41]. Subsequently, Fe^3+^ is degraded to the highly reactive Fe^2+^ under the action of the six-transmembrane protein of prostate 3 (STEAP3). Mediated by divalent metal transporter 1 (DMT1), Fe^2+^ translocates from endosomes to the cytoplasm and then forms an unstable iron pool in the cytoplasm. Part of the Fe^2+^ in the iron pool is stored in ferritin to protect cells and tissues from iron mediated damage, while another part of the Fe^2+^ can be pumped out of the cell through ferroportin on the cell membrane. Under normal conditions, intracellular iron concentrations remain stable.

Once the balance is broken, such as iron when overloaded, excessive Fe^2+^ will be produced within the cell; then Fe^3+^ and hydroxyl radicals can be directly catalyzed by the Fenton chemical reaction. Hydroxyl radicals are the most unstable oxygen free radicals in the human body, and they are also highly active lethal ROS. They get electrons easily from other molecules to cause lipid peroxidation and ferroptosis. Fe^3+^ can in turn be reduced to Fe^2+^ by the superoxide radical (O_2_-) reaction, which is also known as the Haber-Weiss reaction [38]. Under stress conditions, ferritin self-degrades into Fe^2+^ by the process of iron autophagy and then induces ferroptosis.

Increasing iron intake by TfR1, reducing iron excretion through ferroportin, or reducing the stable iron by self-degradation of ferritin all can cause intracellular iron overload to stimulate oxidative damage and ferroptosis [18]. In addition, recent studies have found that iron overload also induces the noncanonical ferroptosis pathway (such as the concentration of ferric chloride, heme, hemin, or ammonium ferrous sulfate), which is sufficient to cause ferroptosis [42, 43]. Iron export protein CDGSH iron sulphur domain 1 (CISD1) in the mitochondrial membrane can reduce the accumulation of iron and the production of ROS in the mitochondria, thereby inhibiting the occurrence of ferroptosis. Meanwhile, voltage-dependent anion channel 2 (VDAC2) and VDAC3, components in the outer mitochondrial membrane, are thought to be the targets of Erastin, which can regulate mitochondrial function through ferroptosis [43]. Nevertheless, the role of mitochondria in ferroptosis remains controversial [44]. By reducing the iron overload, the iron chelators (DFO, desferrioxamine mesylate, and ciclopirox olamine) can inhibit Erastin-induced ferroptosis [45].

#### 2.1.2. Lipid Metabolism Pathway and Accumulation of Lipid Peroxide

Also important for the occurrence of ferroptosis is the creation of ROS and the accumulation of lipid peroxides. For getting into the phospholipids (PLs), the newly synthesized fatty acids must transform the long chain fatty acids into coenzyme A (CoA) [38]. An important regulatory enzyme in the execution of ferroptosis [46], Acyl-CoA synthetase long chain family member 4 (ACSL4) can catalyze the acylation reaction of arachidonic acid (AA) and adrenic acid (AdA) [46]. Catalyzed by lysophosphatidylcholine acyltransferase 3 (LPCTA3), acetylation products combine with phosphatidylethanolamines (PE) into the membrane phospholipid to produce PE-AA and PE-AdA [47]. ACSL4 and LPCTA3 can make the cell membrane rich in sensitive PUFAs and lipoxygenase (LOX), especially 15-lipoxygenase (15-LOX) [39], then oxidize PUFAs (PE-AA and PE-AdA) into lipid hydroperoxides in the form of ferroptosis signal PE-AA-O-OH or PE-AdA-O-OH [48].

When a large amount of Fe^2+^ gathers in the cytoplasm, it can make lipid hydroperoxide form toxic lipid ROS and cause cell damage. These lipid radicals will seize the electrons near the PUFAs, launch a new round of lipid oxidation reaction, and cause more serious oxidative damage. Genetic or pharmaceutical inhibition of ACSL can block the erroptosis pathway [47]. LPCTA3 is a specific substrate of PE, and its deletion can reduce ferroptosis induced by GPX4 inhibitor RSL3 [46]. Vitamin E can inhibit the occurrence of ferroptosis through 15-LOX [48]. Phosphatidylethanolamine-binding protein (PEBP1) can increase the binding of LOX15 and PUFAs in the cell membrane and promote the occurrence of ferroptosis [48].

#### 2.1.3. Execution of Ferroptosis

**Figure 2:**
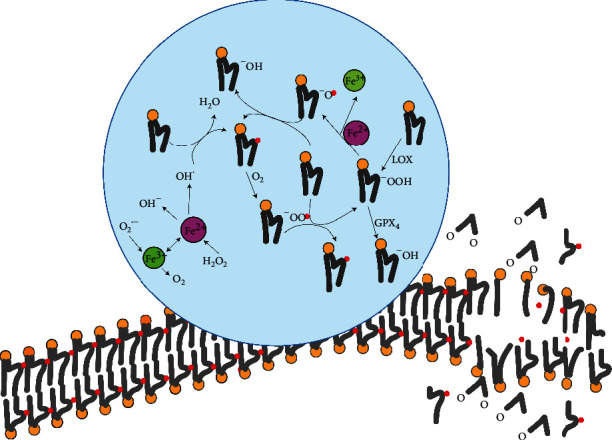
Lipid ROS attacks phospholipid bilayer membrane which is a cascading free radical chain reaction as shown inside the circle, and the productions (PUFA-PLs, 4-HNE, and MDA) reduce membrane stability and increase its permeability to cause cell death.

Ferroptosis is inextricably linked to the generation and function of ROS, but reports on how it targets the cell membrane and executes ferroptosis are rare. The involvement of PUFAs makes the role of free radicals more complex. How it is integrated into the PLs after acylation and how to produce lipid free radicals determine the advance of ferroptosis. It is impossible for free PUFAs to enter the ferroptosis pathway; they must be activated or incorporated into the PLs to participate in this lethal process [40]. Whether PUFAs will be integrated into the PLs in different ways (such as phosphatidylinositol, phosphatidylcholine, or phosphatidylethanolamine PE) depends on the length of the carbon chain and the degree of unsaturation [49]. Among them, it is essential that acylated chains (COA) of AA (C20:4) and AdA (C22:4) integrate with PE for ferroptosis to occur [47]. ROS attack the mammalian phospholipid bilayer membrane in a cascading free radical chain reaction, in which different lipid radicals participate in besides the hydroxyl radical (Figure 2).

There are two main pathways of lipid ROS in ferroptosis (Figure 2): (1) in the nonenzymatic lipid peroxidation pathway, free radicals (^·^OH) capture the hydrogen ions of PUFAs in the plasma membrane and form a phospholipid free radical (PL) centered on carbon atoms. The PL^·^ acts with the oxygen molecule (O_2_) to produce the phospholipid hydrogen peroxide radical (PLOO). PLOO^·^ can also capture hydrogen atoms from PUFAs, thus forming phospholipid hydroperoxide (PLOOH) and a new PL^·^, and PL^·^ can mediate a renewed oxidation reaction. This cycle will affect more and more PUFAs in the cell membrane. (2) In the enzymatic lipid peroxidation pathway, lipoxygenase (LOX) is indispensable to this process, and it can catalyze the dehydrogenation of PUFAs to form PLOOH [38]. Next, PLOOH is decomposed into the alkoxyl phospholipid radical (PLO) by the presence of Fe^2+^, which can also attack other PUFAs to trigger a chain reaction of lipid peroxidation. On the other hand, PLOOH can be decomposed to 4-hydroxynonenal (4-HNE) and malondialdehyde (MDA), which can deactivate membrane proteins by a cross-linking reaction [38]. PUFA-PLs, 4-HNE, and MDA will reduce the stability of the cell membrane and increase its permeability, thereby resulting in cell death [38].

This is the execution of ferroptosis. Besides GPX4, ROS is also regulated by specific inhibitors of ferroptosis (such as Fer-1 and lip-1), [38, 50], which can prevent the accumulation of PL peroxide and act as a lipophilic reductant or free radical scavenger to restrain lipid peroxidation [27, 51, 52]. Fer-1 has been proven to improve cell death in the cortex striatum slices of Huntington mutant rats, in the periventricular white matter softening cell model, and in the injury test of mouse convoluted tubules [27]. lip-1 can inhibit ferroptosis in GPX4-deficient fibroblasts, in GPX4-depleted mouse models, and in ischemia-reperfusion models [53].

### 2.2. Regulatory Mechanisms of Ferroptosis

#### 2.2.1. Regulation Pathway of GPX4 Based on Glutathione (GSH)

This pathway is an essential regulatory mechanism of ferroptosis, in which GPX4 plays a key role. A selenoprotein synthesized in the presence of selenium (Se), GPX4 is able to eliminate the PLOOH of PUFAs by transforming activated PLOOH into inactivated phosphatidylcholine (PL-alcohol, PLOH), thereby interfering with the chain reaction of free radicals, inhibiting the lipid peroxidation process, and suppressing ferroptosis [38]. Conversely, it was already showed deletion of GPX4 in mice and cells revealed downstream 12/15-lipoxygenase-derived lipid peroxidation and triggered apoptosis-inducing factor-mediated cell death [54], now identified as ferroptosis.

Glutamate/cystine reverse transporter system Xc- on the cell membrane is comprised of the solute carrier family 3 member 2 (SLC3A2) and the solute carrier family 7 member 11 (SLC7A11). The transporter promotes transmembrane exchange of extracellular cystine and intracellular glutamate, and cystine into the cell can be induced to produce cysteine (Cys)—an indispensable substrate for GSH synthesis. As an essential cofactor of GPX4, GSH maintains the activity and expression of GPX4, and both are antioxidants that can eliminate ROS. Ways in which GPX4 decreases ROS and blocks ferroptosis include the following: (1) GPX4 eliminates PLOOH in the chain reaction against lipid peroxidation and converts it into alcohols under the assistance of reduced GSH; (2) GPX4 can also antagonize active Fe^2+^, so that H_2_O_2_ is converted to H_2_O [55]. Raised extracellular glutamate concentration is not conducive to the reverse transport of System Xc-, thus affecting the synthesis of GSH. In the case of cystine deficiency, methionine will produce cysteine through the transsulfuration pathway to secure GSH synthesis.

Erastin and Sulfasalazine induce ferroptosis by inhibiting the transporter activity, while thiosyl ethanol inhibits ferroptosis through increasing cystine transport into the cells to enhance GPX4 activity [9]. In essence, the occurrence of ferroptosis depends on the balance between the oxidation system (Fe^2+^, ROS, etc.) and the antioxidant system (GPX4, GSH, etc.). Under normal circumstances, the two strike a balance in the cell and play a normal physiological role. When encountering injury or stress, the oxidation system will be enhanced (such as active iron, lipid peroxides, and increased iron reactive oxygen production) or the antioxidant system will be weakened (such as GSH depletion and GPX4 inactivation), and the balance will be broken; then the lipid peroxidation and inducement of ferroptosis will be accelerated.

The inactivation of GPX4 mainly depends on the following two mechanisms: (1) in the indirect way, by reducing or consuming Cys, the synthesis of GSH is insufficient, and the lack or depletion of GSH will in turn affect the synthesis and activity of GPX4. These inducers are also known as type I ferroptosis inducers (FINs), such as system Xc- inhibitor, Erastin, Sulfasalazine, Sorafenib, and Glutamate [56]. (2) In the direct ways, several compounds (RSL3, ML162, FINO2, etc.) can directly bind to GPX4 or inactivate it [39, 57–59]; among them, RSL3 can inactivate GPX4 by catalyzing alkylation of selenocystine [39, 57]. Such drugs are also called type II FINs. Insufficiency or inactivation of GPX4 reduces the lipid peroxide repair capacity, which is one of the most important features of ferroptosis [40], and then finally triggers ferroptosis [57]. The latest research indicates that interferon gamma (INF*γ*) released by CD8^+^ T cells can downregulate the expression of two subunits of glutathione antiporter (SLC3A2 and SLC7A11), reduce the uptake of cystine by tumor cells, interfere with GSH synthesis, and affect the production and activity of GPX4, thereby enhancing lipid peroxidation and inducing ferroptosis to have an antitumor effect [60].

#### 2.2.2. Mevalonate (MVA) Regulatory Pathway

The MVA pathway, one of the critical pathways of cell metabolism, regulates cholesterol synthesis and isoprene modification of the small G protein after translation. The pathway takes more than 30 steps in the cytoplasm and mainly includes the following key steps: first, acetyl coenzyme A (Acetyl-CoA) forms 3-hydroxyl-3-methylvaleryl two coenzyme A (HMG-CoA) under the action of 3-hydroxyl-3-methylreductase (HMGCR). The latter can be reduced to MVA by a restriction enzyme. MVA produces isopentyl pyrophosphate (IPP) under the action of a series of enzymes such as mevalonate kinase (MVK). IPP produces farnesyl pyrophosphate (FPP) under the action of farnesyl phosphate synthetase, which produces squalene catalyzed by squalene synthetase (SQS). Then cholesterol is eventually formed under the enzymatic action of squalene cyclase [61].

In the above process, IPP is an important intermediate that can be used for the synthesis of various biological molecules, including cholesterol, coenzyme Q, vitamin K, and heme [62]. IPP and FPP bypass the cholesterol pathway and produce noncholesterol products, such as coenzyme Q_10_ (CoQ_10_) which is a kind of antiperoxide for free radical scavenging, under the action of pyrophosphate synthase (GGPS). Activation of SQS can promote the synthesis of cholesterol in the MVA pathway but reduces the formation of C_O_Q_10_.

The MVA pathway also plays an important role in the maturation of GPX4. In the synthesis of selenoprotein GPX4, selenocysteine (Sec) has to be inserted into its catalytic center to exert its antioxidant activity, and this complex step is attributed to the role of IPP. IPP acts as a donor to promote the formation of isoprene transferase, which catalyzes mutation of the selenocysteine transporting RNA at specific adenine sites [63]. Thus, it promotes the integration of selenocysteine on the GPX4 catalytic subunit, then benefits synthesis and maintenance of the activity of GPX4 [64]. This process can be blocked by FIN56, thereby reducing the expression and activity of GPX4. Meanwhile, FIN56 can activate SQS and promote the MVA pathway to turn to cholesterol synthesis. Therefore, FIN56 can be seen as an inducer of ferroptosis.

Though the MVA pathway is a widely studied metabolic pathway, the newest discoveries have recently caused much attention to be given to it again. In 2019, Doll and other scholars discovered that the expression of the mitochondrial associated apoptosis inducing factor 2 (AIFM2) gene could make up for the function of GPX4 in human GPX4 deletion cancer cells. To avoid confusion, the gene was renamed ferroptosis inhibitory protein (FSP1), and it could inhibit the occurrence of ferroptosis in the absence of GPX4 [65]. Subsequent studies found that to eliminate lipid peroxidation, CoQ_10_ needed to be transformed into lipophilic antioxidant CoQ_10_-H_2_ by FSP1. Then CoQ_10_-H_2_ could remove PLOOH and thus terminate the chain reaction against lipid oxidation and inhibit ferroptosis. The FSP1/CoQ_10_/NAD (P) H pathway was considered to be a parallel and independent ferroptosis inhibiting pathway to the GSH/GPX4 pathway, and the inhibitors of FSP1 (iFSP1) proved to have the ability to promote lipid peroxidation and restrain ferroptosis by blocking the role of FSP1 [65, 66]. Bersuker et al. [67] supported the above findings and indicated it may enhance the antitumor activity of chemotherapeutic drugs by inducing ferroptosis.

#### 2.2.3. Other Regulatory Pathways in Ferroptosis

In addition, there are other pathways that participate in the regulation of ferroptosis. For example, the apoptosis molecule P53 inhibits transporter activity by decreasing the expression of system subunit SLC7A11, reducing cystine intake, decreasing GSH synthesis, inhibiting GPX4 activity, and thus increasing lipid ROS and causing ferroptosis [68]. Activation of the mitogen activated protein kinase (MAPK) pathway can induce ferroptosis in cancer cells, for instance, blocking RAS/RAF/MEK/ERK in the MAPKs family can inhibit ferroptosis brought about by Erastin in RAS mutant cancer cells [69]. By inhibiting the RAS/RAF/ERK signaling pathway, ferroptosis inhibitor U0126 can protect neurons and promote axonal regeneration, thereby repairing the spinal cord and reducing the formation of a glial scar in the injured area [70].

When being translocated into cells, glutamine in the intercellular fluid undergoes a series of chemical reactions that decompose it into glutamate, aspartic acid, alanine, and other intermediates. Studies have shown that the reactions play an important role in the development of tumors [71]. As a key intermediate in the catabolism of glutamine, L-glutamine is shown to further induce the formation of ROS and eventually ferroptosis [72].

### 2.3. The Role of Selenium in Ferroptosis

Selenium was first discovered in 1817. In the early days, it was thought to be a highly toxic micronutrient and even a carcinogen related to hair loss, diarrhea, and vomiting. However, subsequent animal studies showed that selenium was an essential trace element in mammals and could prevent liver necrosis caused by vitamin E deficiency [73]. Since then, scholars have come to realize the vital role of selenium in mammalian life. Selenium plays a role in mammalian life mainly in the form of selenoprotein. The human proteome has 25 different selenoproteins, of which GPX4 is the most important and most widely studied.

As mentioned earlier, GPX4 is an important molecular marker for the mechanism of ferroptosis. Several pathways, including Xc-/GSH and MVA, rely on it for regulation. Meanwhile, GPX4 is a very important selenoprotein closely related to selenium. On the one side, selenium can protect GPX4 from irreversible inactivation [74]. After replacing the selenium contained in the GPX4 of the mouse model with sulfur, the mice survived for no more than 3 weeks because of neurological complications [75]. On the other side, selenium can drive GPX4 transcriptional expression to protect neurons from oxidative stress and inhibit ferroptosis. The close relationship between selenium and GPX4 appeared to be not limited to neuronal cells. When comparing liver specific Trsp and GPX4 knockout animal models, scholars found that liver-specific GPX4 deletion mice showed a more severe phenotype than Trsp knockout mice, which died 1 to 2 days after birth, while Trsp knockout mice survived longer than 3 months [76].

The in-depth understanding of the transcription and translation mechanism of selenoprotein has enhanced the study of GPX4 expression and activation regulation. Early studies showed that the active center of selenoprotein GPX4 was Sec, and its integration into GPX4 required the transport of specific mature tRNA (tRNA^[Ser]Sec^), which was attributed to the same UGA of the Sec genetic codon and stop codon. The translation process aided by tRNA^[Ser]Sec^ can avoid the termination codon expression, so that it specifically translates Sec. tRNA^[Ser]Sec^ regulates the Sec translation pathway, thus directly affecting the efficiency of the translation. IPP, the metabolic intermediate generated by the MVA pathway [77], can be isomerized to Dimethyl allyl diphosphate (DMADP), and the latter is the substrate of tRNA isopentenyltransferase 1 (TRIT1). The formation of TRIT1 brings isopentenylation of site 37 of adenylate on tRNA [Ser] Sec subunit [78], producing isopentenyladenosine [63]. The above changes promote this maturation of tRNA^[Ser]Sec^ and ensure the maximized efficiency of the UGA codon translation. Mature tRNA^[Ser]Sec^ can specifically translate Sec and integrate the group into the active center of GPX4 to guarantee its activity.

This process cross links the MVA pathway to the Cys/GSH/GPX4 pathway; in other words, these two pathways can interact through intermediate factors. The side-effects of statins support this process. As an inhibitor of the HMG-CoA reductase, statins block the MVA pathway [77], and long-term use of statins in patients with hypercholesterolemia would decrease selenoprotein synthesis [79] and hinder the generation of GPX4 [80], which would lead to excessive oxidative damage and muscle diseases. The combined use of statins with ferroptosis inhibitors or coenzyme Q could prevent this adverse reaction [81, 82].

In 2018, 200 years after the discovery of selenium, Ingold et al. [64] found that the development of an important interneuron (parvalbumin-positive neurons) in the brain was dependent on the presence of selenium during the development of mice [83]. The researchers created a special mutant mouse model (GPX4^cys/cys^), whose selenium atoms in the GPX4 protein of the body were all replaced by sulfur. Results showed the contents of parvalbumin-positive neurons were greatly decreased, and all the selenium-deficient mice died of fatal epilepsy by 18 days. Additional studies revealed GPX4 is critical to the maturation of mouse parvalbumin-positive neurons after birth. When selenium in GPX4 is replaced by sulfur, the activity of GPX4 decreases significantly and the accumulation of peroxides in the parvalbumin-positive neurons cannot be effectively removed, resulting in the death of a large number of neurons and eventually leading to severe epilepsy and mouse death [64].

Research published in 2019 showed [30] selenium could increase the expression of GPX4 and other selenoproteins by coordinating activation of transcription factors TFAP2c and Sp1, thereby enhancing the resistance of GPX4 to oxidative damage and finally inhibiting ferroptosis in vitro and in vivo studies of intracerebral hemorrhage (ICH). It was the first to show that selenium can block ferroptosis and treated stroke by enhancing the transcriptional adaptability of neurons. In an in vitro study, the best concentration of selenium could significantly inhibit heme and HCA-induced ferroptosis to protect neurons and also reduced side effects of drugs. However, the selenium was dose-dependent, its effective concentration window was narrow, and it was easy to overdose. The selenium was usually absorbed into the cells in the form of sodium selenite [84, 85], and it was used for the synthesis of Sec. In the ICH model, sodium selenite was administered by intracerebroventricular injection [86, 87], which was easily infected, and the clinical operation was inconvenient. More crucial was that the scope of the safe concentration window of selenium was narrow, so its clinical use was limited. For the sake of settling the matter, Alim et al. developed a more innocuous and effective peptide selenium (Tat SelPep), which can be used through intraperitoneal injection [30].

## 3. Progress in Research on Ferroptosis in SCI

SCI is an acute traumatic disease of the central nervous system, which always leads to different levels of motor, sensory, and autonomic dysfunction, and thus reduces the activity of daily living and the quality of life. The mortality and disability rate of SCI is high, and a sizeable proportion of patients has permanent dysfunction and cannot take care of themselves. As rehabilitation therapy is restricted and its curative effect is unsatisfactory, treatment of SCI is one of the medical problems that have troubled mankind. The reason why SCI is difficult to treat is not only the irreversible characteristics of nerve injury but also the mechanism of SCI which is a complex process which can be divided into primary injury and secondary injury. Primary injury refers to mechanical contusion or extrusion occurring at the moment of injury, which depends on the twisting force, compression force, and nerve transection degree, and is often irreversible. Secondary injury is the continuation and development of the primary injury, which is a complex cascade amplification reaction involving multiple mechanisms, including spinal cord tissue edema, oxygen free radical injury, inflammatory reaction, calcium overload, ion channel damage, glutamate toxicity, mitochondrial dysfunction, blood spinal cord barrier destruction, axonal demyelination, necrosis, and apoptosis, and its harm even exceeds that of the primary injury [88]. Due to the irreversibility of the primary injury, we should focus our efforts on dealing with the cascading secondary injury to prevent spontaneous neuronal death and degeneration and to stimulate axonal regeneration.

Does ferroptosis exist in SCI? Some scholars have found that the ferroptosis markers in the spinal cord tissue of SCI rats were obviously changed, and using transmission electron microscopy, they also observed changes in the mitochondria characteristic of ferroptosis, thus confirming that ferroptosis plays an important role in SCI [35]. After SCI occurred, the spinal cord bled profusely, red blood cells accumulated, cells broke up, and hemolysis occurred, and there was a local iron overload. In addition, stress activated a large amount of ROS and increased the excitatory toxicity of glutamate. All these factors induced the occurrence of ferroptosis.

**Figure 3:**
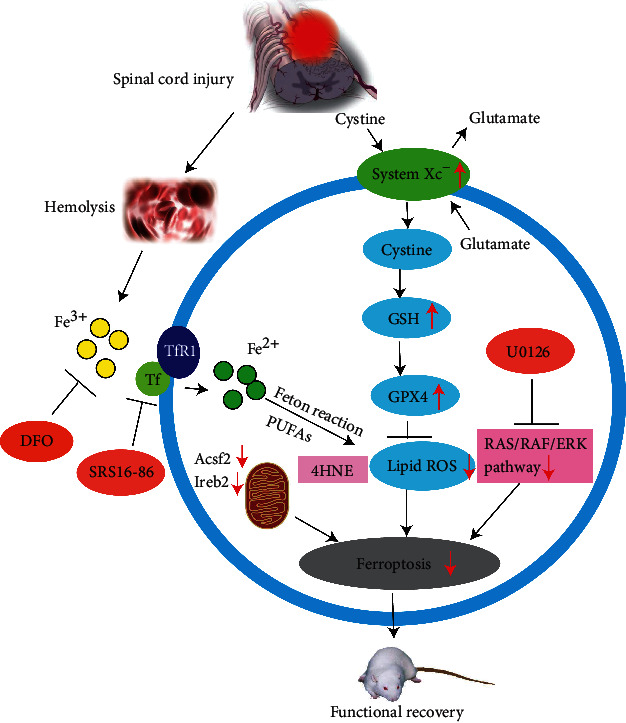
Summary of the role of ferroptosis in spinal cord injury.

Subsequent studies confirmed that ferroptosis was an important cause of the serious consequences of secondary injury after SCI, and DFO could promote the recovery of motor function in SCI rats by inhibiting ferroptosis [35]. Galluzzi et al. [89] added ferrous ions to the culture dish of spinal nerve cells and observed that the amount of lipid peroxidation metabolites was proportional to the level of iron and positively correlated with neuronal inactivation. Zhang et al. [36] discovered that intraperitoneally injecting the ferroptosis inhibitor (SRS16-86) in SCI rats effectively reduced the ferroptosis-related metabolite 4-hydroxylnonenal (4HNE) and upregulated GPX4, xCT, and GSH, thereby reducing the redox damage after SCI. The mitochondria morphology came closer to normal, and more mitochondria crest could be seen after the intervention. The inhibitor could also decrease the astrocyte proliferation, reduce the inflammatory reaction, and increase neuronal survival [36]. Hao [90] also confirmed this phenomenon. Later activation of the extracellular regulated protein kinase (ERK) pathway is considered to be one of the ferroptosis signs, and the blocking of the RAS/RAF/ERK pathway by the use of the ferroptosis inhibitor U0126 could inhibit astrocyte proliferation, protect neurons, and promote axonal regeneration, thereby restoring SCI and reducing the formation of glial scar in the damaged area [91]. The role of ferroptosis in spinal cord injury is summarily described in Figure 3.

## 4. Existing Problems and Prospects

Although considerable progress has been made in the study of ferroptosis, there are still many problems to be solved. For instance, what is the detailed mechanism of iron metabolism starting and subsequent oxidation reactions? The answer may help to prevent secondary injury caused by ferroptosis in SCI from the source. The newly discovered FSP1/CoQ_10_/DADH pathway is considered to be a parallel and independent ferroptosis inhibiting pathway to the GSH/GPX4 pathway, and what is the distinctive mechanism of this pathway? How can FSP1 enter the cell membrane and play the role of reducing CoQ_10_ and how can CoQ_10_-H_2_ inhibit lipid peroxidation are still unknown. Selenium intervention in ferroptosis remains in the animal experimental stage and far from clinical application.

Previous studies have confirmed that apoptosis after SCI is an important cause of neuronal death [92]. Autophagy has also been described in SCI and how it played a role in neural protecting [93]. Now with the addition of ferroptosis, a newly found cell death pathway, the network of secondary injury after SCI has become more comprehensive and complicated. What is the link between these three cell death pathways in SCI and how do they interact with each other? Recent studies may give some answers [94]. In a rat model of subarachnoid hemorrhage, scholars found autophagy can degrade ferritin in neurons to increase intracellular free iron and then promoted the occurrence of ferroptosis. In addition, researchers have suggested that autophagy is involved in the downstream execution of ferroptosis [95]. Ma et al. concluded that ferroptosis and autophagy were independent of each other, but they were all induced by iron dependent ROS [96]. We need to recognize that all these viewpoints were drawn from other disease animal models. Whether there are consistent changes in SCI and what is the relationship between ferroptosis and other forms of cell death deserve our further exploration.

## 5. Conclusion

As a newly discovered form of cell death, ferroptosis has attracted a lot of attention in recent years and research on it has made immense progress. The mechanisms of ferroptosis include iron metabolism, lipid peroxidation, ROS accumulation, the GPX4 regulatory pathway, the MVA pathway, Se regulation, and the FSP1-mediated pathway. However, research concerning ferroptosis in SCI is still in its initial stage, and there remain a number of problems to be studied.
